# The Future Application of Organ-on-a-Chip Technologies as Proving Grounds for MicroBioRobots

**DOI:** 10.3390/mi11100947

**Published:** 2020-10-20

**Authors:** Haley C. Fuller, Ting-Yen Wei, Michael R. Behrens, Warren C. Ruder

**Affiliations:** 1 Department of Bioengineering, University of Pittsburgh, Pittsburgh, PA 15219, USA; haf54@pitt.edu (H.C.F.); tiw56@pitt.edu (T.-Y.W.); mrb157@pitt.edu (M.R.B.); 2 Department of Mechanical Engineering, Carnegie Mellon University, Pittsburgh, PA 15213, USA

**Keywords:** microrobotics, microbiorobots, organ-on-a-chip, microphysiological systems, drug delivery

## Abstract

An evolving understanding of disease pathogenesis has compelled the development of new drug delivery approaches. Recently, bioinspired microrobots have gained traction as drug delivery systems. By leveraging the microscale phenomena found in physiological systems, these microrobots can be designed with greater maneuverability, which enables more precise, controlled drug release. Their function could be further improved by testing their efficacy in physiologically relevant model systems as part of their development. In parallel with the emergence of microscale robots, organ-on-a-chip technologies have become important in drug discovery and physiological modeling. These systems reproduce organ-level functions in microfluidic devices, and can also incorporate specific biological, chemical, and physical aspects of a disease. This review highlights recent developments in both microrobotics and organ-on-a-chip technologies and envisions their combined use for developing future drug delivery systems.

## 1. Introduction

**Figure 1:**
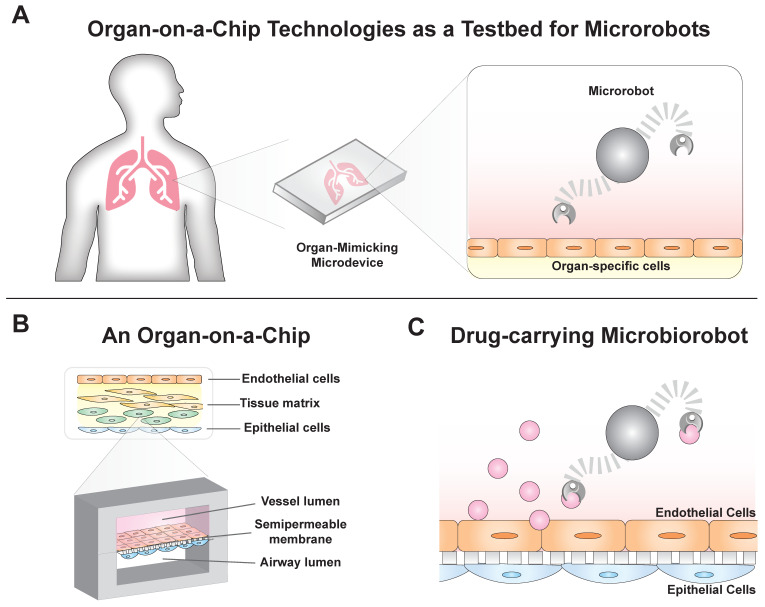
Organ-on-a-chip systems as proving grounds for microbiorobots. (**A**) Organ-on-a-chip technologies have evolved from microfluidics to be able to model physiological conditions and could be used as proving grounds for microrobots before clinical use. (**B**) These systems are 3D models that recapitulate complex tissue anatomy and physiology. (**C**) Drug-carrying microbiorobots could be deployed in these systems to develop precise, targeted therapeutics.

Organ-on-a-chip technologies combine microfluidics with tissue engineering to create robust disease models that mimic in vivo environments. These systems allow for fine control of experimental variables and real-time visualization of cellular responses [1]. As a result, they offer an opportunity for testing microrobots designed to deliver drugs (Figure 1). Many current drug delivery technologies are based on micro- and nanoscale particles that leach drugs into the local tissue environment [2,3,4]. These particles are injected into specific locations or into the body’s blood vessels. In the case of the latter, particles can be designed to home to specific areas by chemically modifying the particle’s surface [5,6]. Drug-laden microrobots can improve upon this functionality by incorporating robotic control of navigation [7], remote control [8,9,10,11], and specific location targeting [12,13], potentially offering a new direction for precision medicine. When loaded with drugs, they have the potential to form new treatments. However, testing these robots in environments representing human physiology will be essential. Here, the utility of organs-on-a-chip is explored for this purpose.

In these systems, organ functions are partially reproduced in microfluidic devices. Cells cultured from a subset of the different tissues that make up an organ can be grown in close proximity (Figure 1B), in structures that mimic the body’s endogenous morphology, which enables cell–cell communication, coordination, and subsequently, the emergence of some organ functions. Researchers can deconstruct disease complexity by introducing environmental perturbations to these systems and observing their effect on disease pathogenesis. Organ-on-a-chip devices have previously been used to investigate blood clot formation in patient samples, and have been integrated into animal extracorporeal circuits to provide real-time monitoring of antithrombotic therapies [14]. As organ-on-a-chip devices are based in microfluidics, it is straightforward to couple these systems to other microscale engineered technologies that have been developed in liquid. As a result, microrobots, often developed in aqueous solutions, can easily be deployed within organ-on-a-chip devices.

One microrobotics application is drug delivery (Figure 1C) and is particularly relevant to a class of microrobots known as microbiorobots (MBRs). These microrobots are partially composed of living or nonliving biological components. In some cases, bioinspired microrobots that do incorporate biological components can also be considered MBRs. In the past decade, MBRs have evolved from drug-laden, flexible, magnetic swimmers [9] to self-folding, multifunctional microrobots capable of untethered (and thus, noninvasive) manipulation for targeted drug delivery [15]. MBRs have uses in nanomedicine; for example, porous nanocapsules are able to respond to different physical conditions such as temperature increases [16,17] or the presence of microwaves [18]. MBR motion has been actuated and controlled using technologies such as customized permanent magnets [19] and programmable motility algorithms [20], as well as strategies that exploit the helical translational action or magnetotactic effect of systems found in nature [13,21]. These different actuation and control strategies are compatible with standard microfluidic and organ-on-a-chip device form factors. As a result, deploying and testing MBR-based drug delivery approaches in organ-on-a-chip devices is the next significant step in assessing the potential of MBRs as delivery systems.

## 2. Organ-on-a-Chip Devices Mimic Physiological Systems

Organ-on-a-chip systems are engineered, biohybrid systems that dynamically integrate mechanical, chemical, and physical inputs to create functionally coupled tissue-like structures in microdevices. They allow fine control over experimental conditions, such as the biochemical and mechanical cues that serve to coordinate tissue development and maintenance. Of particular interest, organ-on-a-chip systems have been shown to provide the individual physical inputs delivered in standard cell and tissue culture yet are also sufficiently adaptable to recapitulate tissue and organ function. Ideally, organs-on-a-chip reproduce organ function so well that they can be considered as alternatives to engineered, three-dimensional, in vitro tissue models or live animal models of disease. In some cases, live animals are insufficient for understanding human disease. For example, Seok et al. showed how mice are poor at mimicking human inflammatory diseases [22].

Organ-on-a-chip development is currently an especially active area of significant research investment. Even prior to this recent focus, researchers had already demonstrated in vitro tissue growth and development in systems partially similar to recent organ-on-a-chip systems. For example, semipermeable membranes that allow different types of cells to be cultured in close physical proximity have existed for decades. In 1997, Fillinger et al. reported the use of a 13 μm-thick semipermeable membrane to investigate the collaborative effect of culturing endothelial cells (EC) and smooth muscle cells (SMCs) together [23]. In this study, SMCs were cultured on one side of a semipermeable membrane and ECs were grown on the opposite side, which allowed for physical contact between SMCs and ECs. Alternatively, each cell type was cultured separately, but connected in a manner that allowed for culture solution exchange between the two cell types. Both types of coculture demonstrated increased SMC proliferation and density—indicators of cell growth and viability—in comparison to cultures of only SMCs. In the case where ECs and SMCs were grown on opposite sides of the membrane, SMCs exhibited spontaneous growth through the membrane’s pores, allowing for contact with the EC layer, which is similar to the actual orientation of these cell types in vivo. A similar semipermeable membrane was later used in the important development of a lung-on-a-chip system [24].

**Figure 2:**
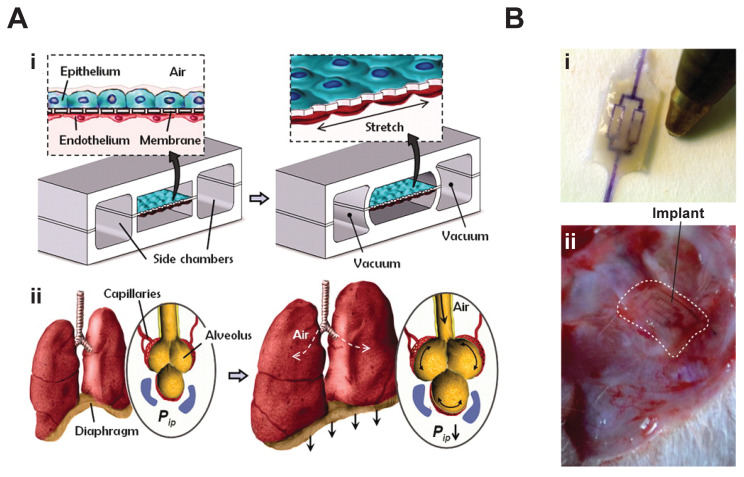
Organ-on-a-chip technologies as engineered biohybrid machines. (**A**) A lung-on-a-chip system was constructed based on (**i**) integrated epithelium and endothelium and integrated respiratory mechanics to mimic (**ii**) the alveolar–capillary interface [24]. (**B**) The (**i**) AngioChip is a porous, biodegradable scaffold based on a branched, fluid channel that mimics vascularized cardiac and hepatic tissue. This biohybrid system mechanically supported perfusion and promoted angiogenesis (i.e., new vessel growth). It also performed in vivo after (**ii**) surgical implantation with rat femoral vessels [25]. Images reproduced with permission from Huh et al. *Science*, 2010 and Zhang et al. *Nature Materials*, 2016.

The 2010 report of a lung-on-a-chip by the group of Donald Ingber was a seminal study and a primary driver of the past decade of organ-on-a-chip research activity. In this system, epithelial cells and endothelial cells were cultured on two sides of an elastomeric, semipermeable membrane (Figure 2A). Epithelial cells formed an epithelium, a barrier between an organ and its environment (e.g., the lining of the lung or gastrointestinal tract); and endothelial cells formed an endothelium, the lining of blood vessels. Here, the researchers combined these tissues to mimic the alveolar–capillary interface found in the lung where blood is oxygenated. The two cell layers formed confluent monolayers in device channels to mimic the flow of blood in the microvasculature and the air transport in the alveolus. Parallel to these channels were two vacuum chambers. When suction was applied to mimic respiratory mechanics, the epithelial-endothelium construct stretched. This mechanical coupling increased the tissue’s response to toxic and inflammatory silica nanoparticles, and stimulated their uptake and transport into the microvasculature, similar to the effect observed in a whole mouse lung. In addition to showing how tissues could be coupled to recapitulate organ function, and how biomechanics can play a critical role, this study also highlighted how laboratory animal use could potentially be minimized by creating organ-on-a-chip devices as replacements.

The integration of biological and mechanical components within organ-on-a-chip systems has allowed for the functional and mechanistic reproduction of physiological structures, including components of the cardiovascular, gastrointestinal, and hepatic systems. In 2015, Yasotharan et al. reported an artery-on-a-chip microfluidic platform to support automated and quantitative immunohistochemical analysis of olfactory artery segments explanted from mice [26]. To recreate the olfactory microenvironment, this research group integrated valves and regulators with a microfluidic device to generate physiological pressure conditions. In another format, Marsano et al. created a beating heart-on-a-chip using microengineered cardiac tissues from both murine- and human-derived sources. This system incorporated uniaxial, cyclic strain, representative of the mechanical environment of the myocardium [27]. Rhythmic stretch and contraction were also utilized by Lee et al. to direct flow through a 3D gastric organoid in order to model gastrointestinal functions in a stomach-on-a-chip [28]. In a vascularized human liver acinus microphysiological system (vLAMPS), Li et al. characterized oxygen zonation and uninterrupted delivery of circulatory support in a microphysiological system by leveraging the hydrophobicity of glass in their device [29]. These various physiological structures provide real-time optical monitoring of complex physiology, demonstrate superior functionality with respect to other in vitro systems, and provide a platform for mechanical and biochemical costimulation.

In some instances, organ-on-a-chip systems have been successfully integrated in living organisms, demonstrating their biocompatibility and their physiological similarity to the endogenous environment. The AngioChip (Figure 2B), developed by Zhang et al. in 2016, features a porous microchannel scaffold and a mechanically tunable matrix resembling a microvascular network [25]. Upon endothelialization (i.e., after a surface becomes covered by a continuous layer of endothelial cells), the scaffold supports a permeable vessel lumen capable of surgical implantation. In addition to enabling remodeling, extravasation, and intercellular crosstalk in an experimental setup, the AngioChip can immediately establish blood perfusion when connected to femoral vessels of murine hindlimbs. It also promotes angiogenesis (i.e., the growth of new blood vessels) within a week following the procedure. More recently, Lee et al. engineered an implantable poly(lactide-co-glycolide) (PLG) scaffold to aid in the investigation of tumor progression and metastasis in hypoxic microenvironments [30]. These microporous PLG scaffolds enable the creation of a readily accessible, subcutaneous site that can be chemically modified to investigate hypoxic regulation, blood vessel formation, and tumor cell migration.

Organ-on-a-chip systems also have demonstrated potential for in vitro drug toxicity screening. Healy and colleagues have improved drug discovery safety and efficacy testing using in vitro systems with the development of their cardiac microphysiological system [31]. In this system, human cardiac tissue, derived from induced pluripotent stem cells (iPSCs), was cultured within the boundaries of a 2 μm-thick, endothelium-like, semipermeable barrier that allows for diffusion of nutrients and drug candidates, which ultimately provided results that were more consistent than cardiotoxicity data produced from human cardiac tissue samples. A series of interconnected tissue culture chambers can allow for interactions between separate cultures to form a single system. As an example, a system developed by Shuler and colleagues used microfluidics and mathematical modeling to predict the pharmacokinetic–pharmacodynamic (PK–PD) effect of drug candidates in interconnected, multiorgan systems [32]. These systems featured multiple cell culture chambers representing liver, tumor, and marrow tissues, connected with fluidic channels to mimic blood circulation. The researchers used this system to screen toxicity responses to a novel anticancer drug. In 2017, Skardal et al. studied systemic, nonspecific effects of a drug using a system that integrated individual tissue constructs with a closed circulatory perfusion loop [33]. Multiple efforts have demonstrated the efficacy of organ-on-a-chip systems for drug safety testing, and thus, they offer a clear opportunity for testing the safety of drug delivery by MBRs.

Organ-on-a-chip systems have been used to recapitulate human physiology in microdevices by incorporating mechanical, chemical, and physical features of native tissue. Highlighted in this section are seminal studies of organ-on-a-chip systems that either demonstrated basic organ function, served as implantable scaffolds for tissue regeneration, or were used as drug toxicity screening tools. Moving forward, organs-on-a-chip that reproduce the native physiological environment could provide an arena in which MBRs can be deployed to assess their drug delivery potential.

## 3. Microbiorobots: Motility in Fluid Environments and Penetrating Tissue Barriers for Drug Delivery

**Figure 3:**
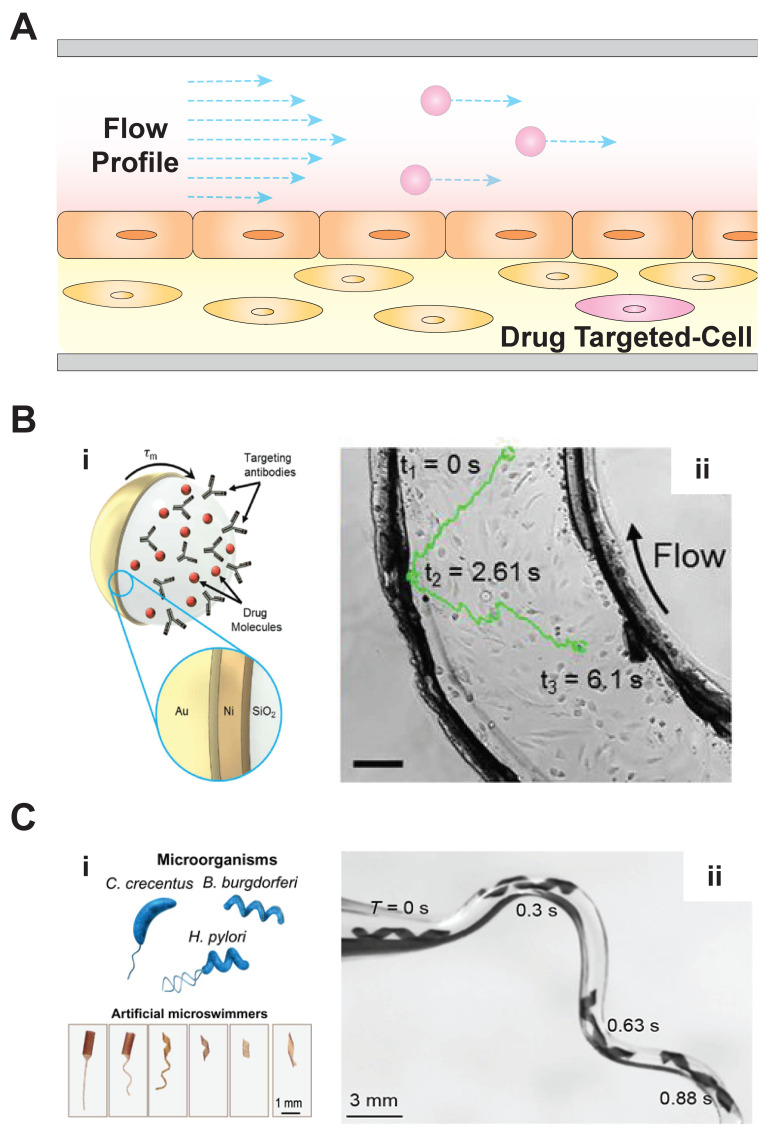
Microbiorobot motility and its relevance to organ-on-a-chip systems. (**A**) Microbiorobots (MBRs) will need to navigate within the laminar fluid regime generated in microvessels in order to precisely deliver drugs to a target. (**B**) Multifunctional microrollers have been engineered with (**i**) antibodies to target and bind cancer cells present in endothelialized surfaces. The microrollers were able to resist (**ii**) disturbances from physiological fluid flow (scale bar, 100 μm) [12]. Images reproduced with permission from Alapan et al. *Science Robotics*, 2020. (**C**) In another approach, (**i**) biologically inspired microswimmers were maneuvered through (**ii**) tortuous glass microchannels, demonstrating the feasibility of MBR delivery in the vasculature [7]. Images reproduced with permission from Huang et al. *Science Advances*, 2019.

Ensuring that MBRs can operate in an organ-on-a-chip’s fluid environment will be critical if these devices are to be used to demonstrate drug delivery efficacy. Fortunately, MBRs are compatible with the fluid flow regimes found in organs-on-a-chip. These devices often aim to reproduce the hemodynamic flow found in the microvasculature. As the circulatory system transports biologics throughout the human body, molecular transport to and from the microvasculature enables oxygen and nutrient exchange. This hemodynamic flow is driven primarily by the heart’s pumping action and regulated by complex cardiovascular mechanisms. When the goal is to precisely target a microscale payload, such as an MBR, to a specific tissue location in the body (Figure 3A), the cardiovascular fluid dynamics can pose a significant hurdle [34]. In particular, as the characteristic diameter of the flow reduces in the transition from large arteries to, ultimately, microscale capillaries, the flow becomes strictly laminar. Organ-on-a-chip systems can reproduce microvascular structures while allowing for fine experimental control over volumetric flow rate, pressure gradients, and flow direction. A range of fluid actuators enables this control, and current examples include syringe pumps [35], peristaltic pumps [36], and gravity-driven flow generators [37]. Microroboticists can use these systems with organs-on-a-chip to create developmental testbeds for studying motility and maneuverability within physiologically relevant conditions.

For their eventual deployment within the body, microrobots must be remotely actuated and their motion must be controlled in order to deliver payloads to the target site of interest. Current approaches for the actuation and motion control of MBRs use light or magnetic fields [8]. Researchers have also harnessed the motility of living organisms to actuate MBRs. One example is the use of magnetotactic bacteria—microbes that naturally synthesize magnetic particles—to deliver drug-laden nanocarriers [21]. As another example of using the capabilities of bacteria to create biohybrid microrobots, bacterial-polymer MBRs embedded with magnetic nanoparticles showed unidirectional chemotaxis under magnetic guidance [10]. Among these different actuation and motion control approaches, magnetic fields are particularly promising for controlling MBRs in organ-on-a-chip systems. Although both light-based and magnetics-based actuation can be used for in vitro systems like organs-on-a-chip, once MBRs demonstrate their utility in these organ-mimicking proving grounds, they will need to be deployed in vivo. Actuation and motion control in the body require the use of energy fields that can penetrate greater depths, through optically opaque tissue. For these applications, magnetic actuation and control will be the optimal systems.

Several medically relevant MBRs have been tested in microdevices with fluid environments inspired by those found in vivo. Sitti and colleagues developed MBRs inspired by the rolling and tumbling motion of immune cells in the blood stream [12]. These immune cells attach to the cells lining the wall of vessels (i.e., endothelial cells) with just enough adhesion to remain in contact with the wall while also allowing the force of the blood flow to cause them to roll along the vessel surface. In their study, Sitti and colleagues reported surface-functionalized, magnetic microrollers (Figure 3B) that achieved translational velocities of up to 600 μm/s across layers of endothelial cells cultured in microchannels. When the team applied a magnetic field, these microrollers demonstrated propulsion opposite to the direction of flow, overcoming shear forces of up to 2.5 dynes/cm^2^. When the team tested the microrollers in culture vessels, they were able to precisely localize the microrollers near targeted cancer cells. In another study, Yu et al. investigated magnetic MBR swarm formation and swimming behavior in biologically inspired fluids of various ionic strength and viscosity. After their surfaces were functionalized to achieve hydrophobicity, the nanoparticle swarms maintained their swim pattern and trajectories in an ex vivo bovine eye tissue sample [38]. Other researchers have reported 3D models of tissue-fluids with potential for integration of organ-on-a-chip techniques and microrobotic interactions. For example, Sitti and colleagues directed stem-cell-carrying MBRs through microchannels using an external magnetic field [11]. Upon reaching the damaged tissue site, the contained stem cells migrated out of the MBRs for localized tissue repair. Bylis et al. demonstrated that chemically propelled microparticles can actively transport functional protein cargo through microfluidic channels perfused with whole blood via lateral propulsion, buoyancy, and convection [39]. This system, which was then deployed in vivo, provided evidence that organs-on-a-chip can be physiologically relevant systems for MBR-directed drug delivery development.

Other microrobots have navigated the fluidic environment using helical, flagella-like tails similar to those found in some bacterial species [7,13,40]. Nelson and colleagues created “adaptive locomotion designs” (Figure 3C) that can be incorporated in microrobots to allow for flexible maneuvering through complex channels that mimic the vessel heterogeneity found within the body [7]. Sitti and colleagues studied the interaction of magnetic helical microswimmers in a microenvironment from an immunogenicity perspective. The response of murine macrophages to these microrobots suggests an opportunity for morphology-dependent design optimization [41]. Wu et al. magnetically actuated helical micropropellers through porcine eyes to deliver drugs to the retina [42]. Xu et al. combined a synthetic microstructure with sperm cells for magnetically guided cargo transport [43]. Bioinspired MBRs capitalize upon the flexibility, propulsion, and biocompatibility of biologically evolved swimming behaviors, which allow for future development to be focused on the merging of precise navigation with efficient and effective drug delivery.

**Figure 4:**
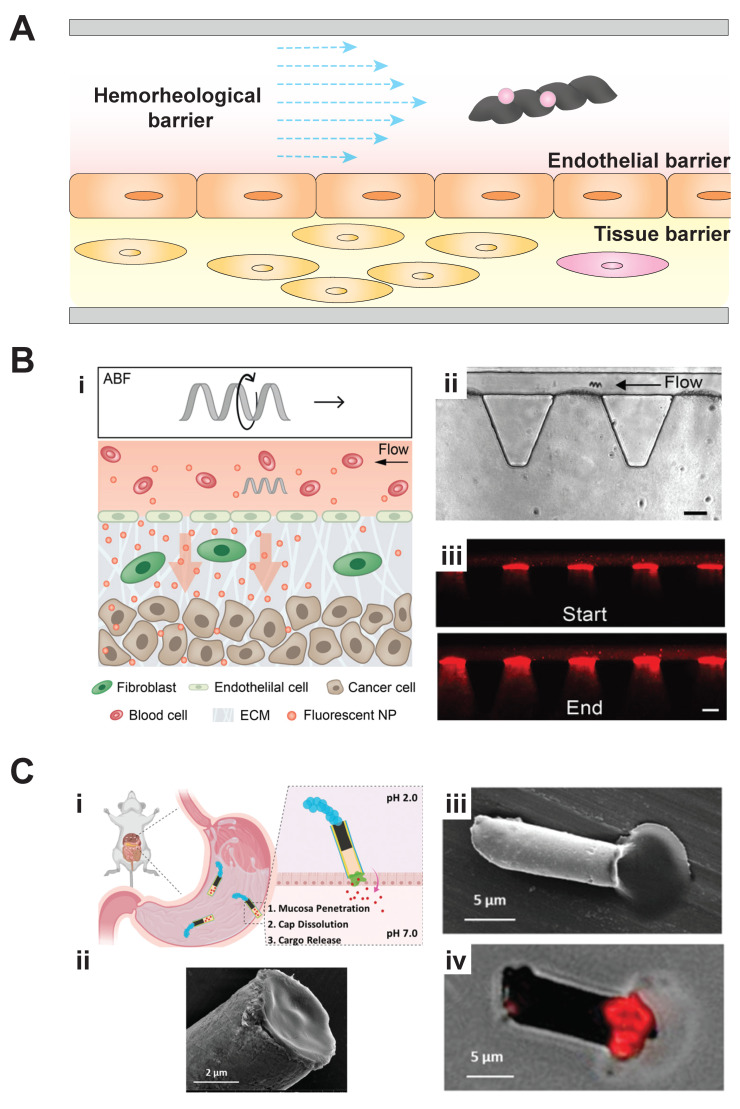
Microbiorobots can penetrate physical barriers in organ-on-a-chip systems. (**A**) Tissue surfaces vary in their diffusive permeability and must be penetrated for drugs to reach their targets. (**B**) Artificial bacterial flagellum (ABF) microbiorobots could enhance (**i**) drug uptake across an artificial vessel–matrix interface in an organ-on-a-chip [45]. (**ii**) Bright-field and (**iii**) fluorescent images revealed an ABF localized along a vessel–matrix interface in the organ-on-a-chip device. Actuation of the ABF facilitated increased nanoparticle transport (scale bar = 50 μm) [45]. Images reproduced with permission from Schuerle et al. *Science Advances*, 2019. (**C**) In another example of enhancing barrier permeability, (**i**) multicompartment microbiorobots demonstrated localized, permeable cargo delivery by utilizing a micromotor as a propulsion system to penetrate tissue surfaces [46]. (**ii**) Polymer-capped-cargo was visualized via SEM imaging and fluorescent imaging to depict (**iii**) dissolution and (**iv**) cargo release [46]. Images reproduced with permission from Esteban-Fernandez de Avila et al. *Advanced Materials*, 2020.

Organ-on-a-chip systems can also replicate the tissue barriers that will need to be crossed by MBR-delivered drugs in future clinical applications. This review envisions MBRs as drug-laden delivery vehicles (Figure 4A). For these drugs to move from the MBR and enter a tissue, they must cross the hemorheological, fluidic barriers found in the vessel, penetrate the endothelium, and diffuse into the surrounding tissue [34]. Fortuitously, several organ-on-a-chip systems have been engineered to understand drug transport across these different barriers at the microscale. For example, Mair et al. used a crawl-based approach in the development of magnetically aligned nanorods in alginate capsules (MANiACs) to form a tumbling robot capable of translational motion across a tissue surface with diffusion-based cargo delivery [44]. Schuerle et al. used helical swimming microrobots to enhance convective transport of fluorescent nanoparticles across a vessel–matrix interface representative of blood vessel extravasation (Figure 4B) [45]. Alternately, Esteban-Fernandez de Avila et al. created an active transport-based system and incorporated a propulsive motor in a multicompartment microstructure to aid in physically penetrating a tissue barrier (Figure 4C) [46]. In this case, the drug of interest was encapsulated in a pH-responsive cap, which only began to dissolve upon penetration of the tissue lining, which exposed the cap to an increased pH. Sufficient propulsion was generated by a zinc-propellant engine. The engine was placed in a microcompartment opposite the payload compartment in a tubular robot chassis. This MBR could then be driven into the targeted area of interest to deliver the payload.

Here, the critical requirement for MBRs to operate in an organ-on-a-chip’s dynamic fluid environment has been described. For future drug delivery applications, microrobots will need to be remotely actuated and precisely controlled to deliver drug payloads. Already, medically relevant MBRs have been tested in physiologically similar fluid environments, and new propulsion systems, inspired by biology, have been developed. These systems for operating in the fluid environment will be critical for exiting blood vessels and penetrating tissue to deliver drugs.

## 4. Organ-on-a-Chip Disease Models for Microbiorobot-Assisted Drug Delivery and a Potential Application

Organs-on-a-chip will also be ideal environments for testing drug-laden MBRs because they can reproduce the environment found in diseased tissues. Thus far, this review has explored both the capacity of organs-on-a-chip for replicating the fluid environment found in tissue as well as their ability to reproduce tissue barriers that must be penetrated by drugs. Yet, their capacity to reproduce specific diseases will be especially important for understanding how MBRs might ultimately be deployed in the body to treat disease.

Following the initial progress in developing organ-on-a-chip systems that replicated different aspects of healthy organ environments, several organ-on-a-chip disease models have been developed. For example, Agrawal et al. developed a musculoskeletal organ-on-a-chip to characterize mechanical strain generated by multinucleated, skeletal muscle bundles [47]. This system was then used to evaluate the effect of dose-dependent muscle injury in response to cardiotoxin. Griffith, Wells, and colleagues probed dormant and metastatic tumor states using hydrogel scaffolds of varied stiffness, representative of healthy and diseased states, in a hepatic microphysiological model [48,49]. While endothelialization of microchannels has often been used to demonstrate the construction of a minimal vasculature for organs-on-a-chip, [12,25,50], Jain et al. expanded upon this approach to study thrombus formation in response to increased physiological shear stress in small volumes of whole blood [51]. The previously mentioned lung-on-a-chip developed by Huh et al. combined bilayer tissue coculture with mechanical stimulation [24], and has been used as a disease model for pulmonary edema by demonstrating vascular leakage in response to cytokines [50,52]. Similar to lung-on-a-chip models, gut-on-a-chip systems have incorporated mechanical forces; in these cases, to mimic the peristaltic motion of the gut. In one example, Ingber, Collins, and colleagues recapitulated the structure of intestinal villi in an organ-on-a-chip to study inflammatory bowel disease [53]. In an effort to investigate cellular crosstalk between the gut and liver, Chen et al. studied bile acid metabolism in a coculture system with both healthy and inflammatory states. Their results were more consistent with in vivo observations when compared to standard in vitro models [54].

Pulmonary hypertension (PH) is an example of a disease where current treatments are limited, and therefore new therapeutics, such as the proposed drug-laden MBRs, should be developed for its treatment. Here, this potential application will be explored to illustrate the key steps in developing an organ-on-a-chip as a proving ground for MBR-based therapies. PH is characterized by narrowed, hardened, and obstructed pulmonary arterioles resulting in an increased mean arteriole pressure [55]. This persistent, increased pressure causes the heart to grow larger and become weak, resulting in irregular perfusion of the lungs and ultimately leads to heart failure [55,56]. Symptoms include chest pain, fatigue, shortness of breath, and swelling in the abdomen or lower extremities. Pulmonary hypertension (PH) will likely remain incurable and fatal without improved experimental model systems that reproduce the cellular, tissue, and organ environment of pathogenesis [57].

Several steps would be needed to create PH-on-a-chip and test MBRs within it. First, a system similar to the Huh et al. [24] device previously described in Figure 2A could be developed to incorporate the key cell types involved in PH [57,58,59]. As their initial lung-on-a-chip system incorporated a vessel lumen, its form provides a starting point for developing a PH arteriole-on-a-chip. In place of the semipermeable membrane used in the Huh et al. system, an extracellular matrix (ECM) layer embedded with SMCs and fibroblasts would be incorporated to recapitulate the medial layer of the vascular wall. During PH pathogenesis, this wall is anatomically remodeled by cellular processes. This improved, living membrane would be held between the epithelialized airway lumen and the endothelialized vessel lumen using a micropillar cage. An example of such a caging system was developed by Kamm, Asada, and colleagues [60], and used by Schuerle et al. [45] as previously mentioned (Figure 4B (ii)). In such devices, the micropillars create surface tension at the air–liquid interface to cage the ECM contents within its boundaries.

**Figure 5:**
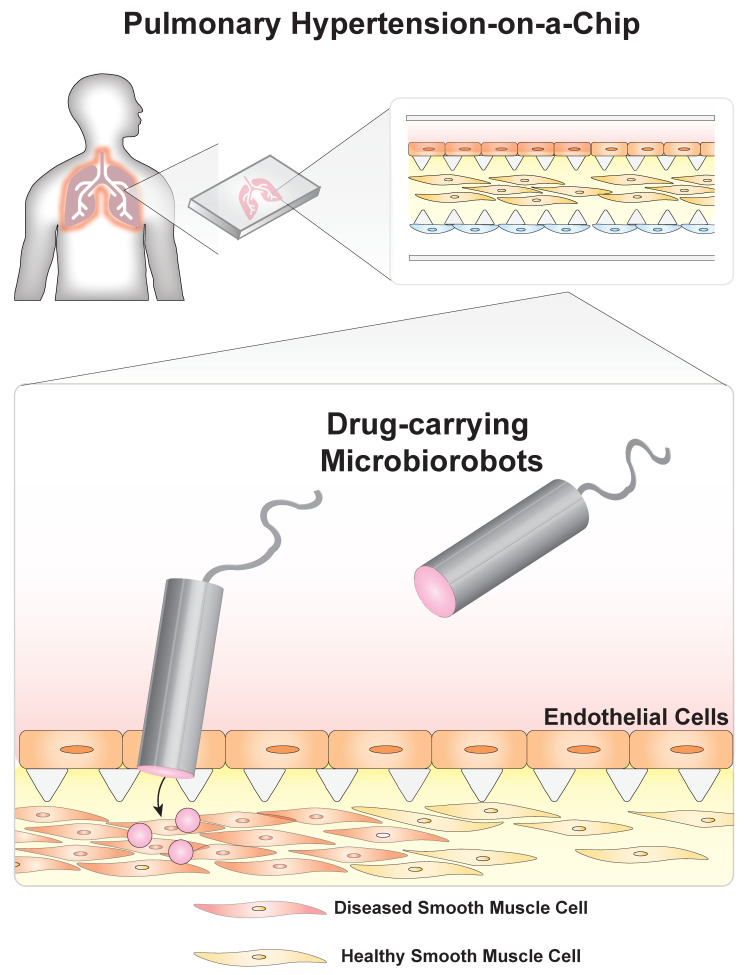
A potential application of MBRs in organs-on-a-chip: treating pulmonary hypertension. The PH-on-a-chip model would incorporate the key cell types (e.g., smooth muscle cells, SMCs) involved in disease progression in an anatomically similar format. For examples, SMCs embedded in extracellular matrix (yellow) could be caged between micropillars (white triangles). This device is inspired by a previously published device used by Schuerle et al. [45]. Next, a magnetically actuated microbiorobot carrying Cpd22, an inhibitor of SMC-driven remodeling [61], could be engineered to maneuver within an endothelialized lumen representing the vasculature. By employing a SMC-sensitive polymer cap [46], the robot would release the inhibitor upon contact with SMCs.

In order to initiate pulmonary hypertension, the system would be probed with different stimuli suspected of triggering pathogenesis (e.g., mechanical and biochemical stimuli) [56]. The PH-on-a-chip model would be expected to withstand mechanical stimulation, such as increased shear stress or intravascular pressure, due to the caging effect imposed by the micropillar supports [60]. Biochemical stimuli could be introduced via a peripheral inlet. MBRs, carrying pharmaceuticals expected to alter disease pathogenesis, would then be introduced to the system (Figure 5). One candidate drug for the MBRs is Cpd22, a signaling inhibitor that has shown promise as a potential therapeutic for reducing vascular remodeling in existing PH experimental models [61]. As this inhibitor has only been shown to have an effect on smooth muscle cells, the organ-on-a-chip’s ECM would need to be penetrated. Here, a compartmentalized MBR would be used. It would contain a cargo-carrying cap, similar to the previously described work of Esteban-Fernandez de Avila et al. (Figure 4C) [46]. For in vivo applications, the MBR’s cargo-carrying cap could even be functionalized to target the endothelial lining of the pulmonary vasculature, and would only release the contained Cpd22 upon detection of chemical markers indicating penetration of the extracellular matrix and localization with the fusion with the contained SMCs [45]. The proposed integration of a multifunctional microbiorobot with a customized PH-disease-model-on-a-chip would be useful both for greater understanding of PH pathogenesis and in developing new, MBR-based treatments for PH.

## 5. Future Directions

**Figure 6:**
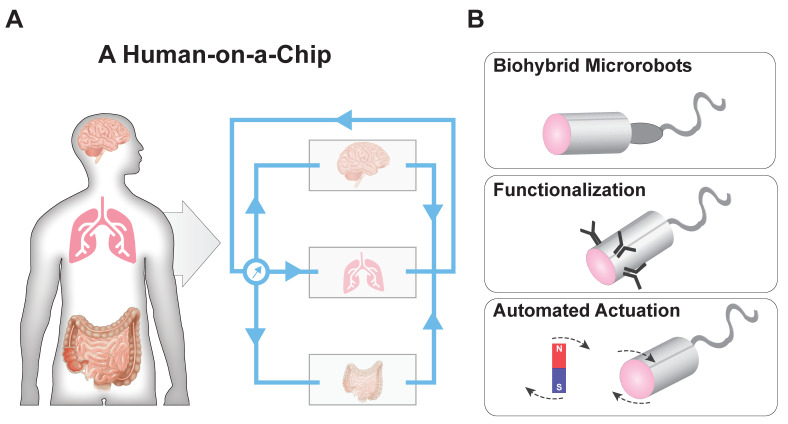
Future directions for microbiorobot drug delivery in organ-on-a-chip systems. (**A**) Plug-and-play human-on-a-chip systems could be customized to monitor systemic effects of drug-laden MBRs, such as changes in the brain, lungs, and gut. (**B**) Using these systems, next generation microbiorobots could be developed to optimize motility, precision, and drug efficacy. Examples of features for customizing microbiorobot would include functionalization with biological components [71]; specific recognition and binding components for targeted delivery [12]; and automated, multiaxis magnetic actuation [72].

Both the organ-on-a-chip and MBR fields have experienced exciting progress over the past decade, and combining these technologies presents a significant opportunity for developing new medical technologies (Figure 6). The current state of the art of integrating microrobots within organs-on-a-chip is demonstrated by the previously discussed work of Schuerle et al. (Figure 4B) [45]. Here, the future of each field is discussed along with their potential for future integration.

The future of organ-on-a-chip research will be in the development of human-on-a-chip systems. To that end, Zhang et al. reported the creation of a compartmentalized, microfluidic cell culture system that mimicked the interaction of organ systems within the body [62]. This system was used to culture liver, lung, kidney, and adipose tissue in individual microenvironments under a common cell culture media. Griffith and colleagues have created 4-, 7-, and 10-organ-like fluidic systems connected via a mixing chamber [63]. In an attempt to increase the throughput of these microphysiological systems, parallel multiorgan experimentation using array formats are gaining traction [64,65], such as the PREDICT-96 platform developed by Tan et al. [66] and the microwell-based, liver-on-a-chip developed by Khademhosseini and colleagues for modeling nonalcoholic fatty liver disease [67]. Along these lines, Lee et al. utilized a pumpless, multiorgan approach combined with mathematical modeling to illuminate the systemic mechanism of action of different drug candidates [68]. Ingber and colleagues utilized liquid-handling robotics to individually culture and analyze eight-organ-on-a-chip systems connected by endothelialized microfluidic channels [69]. Their completely automated system was able to monitor, supplement media, and collect samples in a perfused multiorgan system. Healy and colleagues developed a plug-and-play system—the μOrgano system [70]—which allowed independent organ-on-a-chip systems to be integrated. These types of human-on-a-chip systems will allow for disease-specific understanding of system responses.

As human-on-a-chip systems are realized, next-generation MBRs will be developed for deployment in these devices. These new, drug-laden MBRs will incorporate advanced mechanisms for propulsion and mechanical flexibility to navigate in dynamic fluid environments. Biocompatibility of material components and MBR shape and motility will be required to achieve reliable and noninvasive drug delivery at target sites. Autonomous and automated control will be essential in clinical applications for local sensing and reorientation within optically opaque tissue environments. Automated, multiaxis stability and actuation systems will enable high-precision motion control [72,73]. User-friendly, optics-based motility algorithms that steer robotic systems to within 10 μm of their targets [20] will be employed within organ-on-a-chip systems. Additionally, biohybrid systems that can engage and interact with biological systems for niche treatment options—such as the sperm-driven micromotor for gynecological health developed by Xu et al.—will increase biocompatibility and minimize pathogenic side effects, to speed drug development and encapsulation [71]. In the future, we expect to see the scientific advancement of automated, biohybrid MBRs acting within integrated organ-on-a-chip devices. These systems will serve as proving grounds for new, multifunctional, robotic drug delivery systems for the prevention and treatment of disease.
